# The evolving definition of salivary gland stem cells

**DOI:** 10.1038/s41536-020-00115-x

**Published:** 2021-02-01

**Authors:** Cecilia Rocchi, Lara Barazzuol, Rob P. Coppes

**Affiliations:** 1 Department of Biomedical Sciences of Cells and Systems, University Medical Center Groningen, University of Groningen, 9713 AV Groningen, The Netherlands; 2 Department of Radiation Oncology, University Medical Center Groningen, University of Groningen, 9700 RB Groningen, The Netherlands; 3 Present Address: Centre for Regenerative Medicine, Institute for Regeneration and Repair, The University of Edinburgh, Edinburgh BioQuarter, 5 Little France Drive, Edinburgh, EH16 4UU UK

**Keywords:** Regeneration, Adult stem cells

## Abstract

Dysfunction of the salivary gland and irreversible hyposalivation are the main side effects of radiotherapy treatment for head and neck cancer leading to a drastic decrease of the quality of life of the patients. Approaches aimed at regenerating damaged salivary glands have been proposed as means to provide long-term restoration of tissue function in the affected patients. In studies to elucidate salivary gland regenerative mechanisms, more and more evidence suggests that salivary gland stem/progenitor cell behavior, like many other adult tissues, does not follow that of the hard-wired professional stem cells of the hematopoietic system. In this review, we provide evidence showing that several cell types within the salivary gland epithelium can serve as stem/progenitor-like cells. While these cell populations seem to function mostly as lineage-restricted progenitors during homeostasis, we indicate that upon damage specific plasticity mechanisms might be activated to take part in regeneration of the tissue. In light of these insights, we provide an overview of how recent developments in the adult stem cell research field are changing our thinking of the definition of salivary gland stem cells and their potential plasticity upon damage. These new perspectives may have important implications on the development of new therapeutic approaches to rescue radiation-induced hyposalivation.

## Introduction

Adult salivary glands, like every other tissue and organ in our body, preserve their functionality by maintaining homeostasis, a balance between cell death and cell replacement, which is strictly regulated by adult resident stem/progenitor cells capable of self-renewal and differentiation into mature tissue lineages. Although the function of salivary glands is not a necessity for human survival, the dysfunction of this organ due to radiotherapy treatment of head and neck cancer, leads to long-lasting detrimental side effects. Such side effects, which include difficulties swallowing (dysphagia), eating, and speaking and an accelerated tooth decay and dental caries as well as an increase in fungal and bacterial infections of the oral cavity, can drastically reduce the quality of life of patients^1–4^.

Consistent with the need for new therapeutic approaches that will provide long-term solutions to restore salivary gland function and together with the knowledge that radiotherapy treatment leads to a loss of regenerative potential^3,5^, there has been an increased focus on identifying the stem/progenitor cell populations and the niche signaling pathways that regulate their behavior during tissue homeostasis and regeneration^6–9^.

In this review, we address the challenge of identifying resident adult stem cells, as well as the role they play within the salivary gland during homeostasis and regeneration. Initially presenting salivary gland stem/progenitor cells within the context of the quiescent, multipotent “traditional” stem cell definition, we highlight questions within the field and provide evidence of how recent developments in the adult stem cell research field are changing the perception and quest for identifying salivary gland stem cells from a strictly phenotype-based approach to a more functional approach.

## Classical stem cell definition: the hard-wired dogma of the hematopoietic stem cell (HSC) as template for all other stem cells

Initial attempts to identify stem cells in a relative unexplored tissue, such as the salivary gland, have historically relied on the “stem cell dogma” based on the well-characterized multipotent HSC system^10^. The rarity, the quiescent state, and the ability of HSCs to asymmetrically divide are characteristics that served as a template for all studies aiming to characterize adult stem cells in most mammalian tissues. These characteristics guarantee, on one hand, the “self-renewal and long-lived permanence” of the tissue, and on the other hand, ensure a unidirectional differentiation of well-characterized progenitors along the hierarchical tree until final differentiation is reached^11,12^.

However, can we apply this template based on the only non-solid fast turnover tissue in our body (the hematopoietic system) to solid tissues that differ in size, morphology, physiology, constitution, function, and stressors to which they are exposed to during life? Starting from the HSC point of view, the simplest definition of a stem cell in adult mammalian tissue is a slow-cycling cell that, under homeostatic conditions, limits the number of consecutive divisions to minimize DNA replication errors. In this view, the differentiated cells of a given tissue are derived from transient amplifying progenitors rather than directly from the “primitive” stem cells sitting at the apex of the hierarchical tree. Cells with a low proliferative activity are experimentally defined by the ability to retain chromatin labels, such as 3H-thymidine, bromodeoxyuridine, and histone–green fluorescent protein fusion protein, for an extended period of time and are therefore termed label retaining cells (LRCs).

While retention of nuclear labels essentially defines the proliferative history of cells, studies based on long-term pulse-chase experiments and on the repair of radiation-induced damage in epidermis and intestine^13–15^ showed that LRCs were spatially segregated in these tissues. This led to the proposal that cell cycle characteristics and spatial organization of these cells could describe their identities: slow cycling cells are stem cells (located in the basal layer, in a protected position) and fast cycling cells represent the transient amplifying cells that terminally differentiate after a finite number of divisions following a unidirectional stream starting from the basal layer^14,16,17^. Although the search for novel stem cells based on quiescence is complicated, also considering the fact that the majority of adult cells are not dividing, studies on LRCs (and therefore defining the quiescent state as a “stem cell trait”) were successfully applied in the quest to uncover adult stem cells in several tissues, such as the hair follicle^18^, skin (reviewed in^19^), sweat glands^20,21^, teeth^22^, pancreas^23^, and intestine^24^.

Similar to liver, prostate, and lung, the salivary gland belongs to a group of tissues in our bodies with a relatively slow turnover (>60 days)^25^. These tissues increase proliferation in response to damage, in order to replace the lost cells, to then go back to their low-level maintenance when homeostasis is restored.

## The quest for salivary gland stem cells

**Fig. 1:**
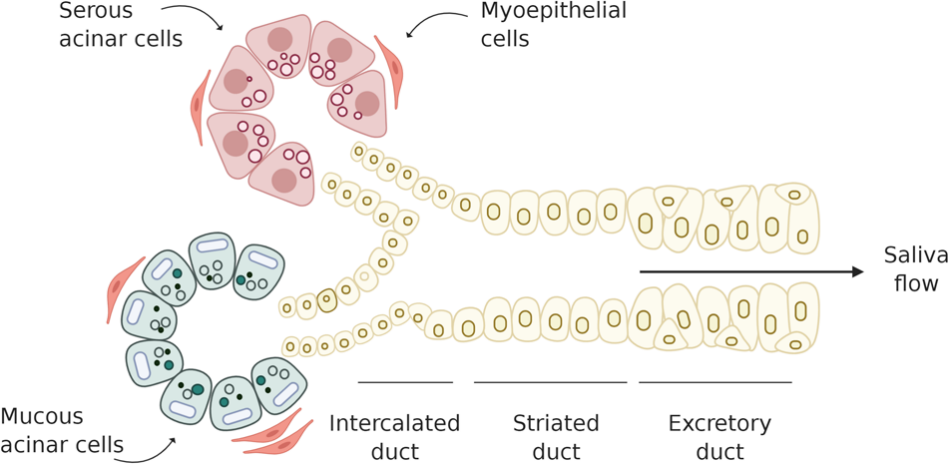
Schematic representation of a generic salivary gland structure. The salivary gland epithelium is composed of two types of saliva-producing cell types, serous acinar cells and mucous acinar cells. Myoepithelial cells surrounding the acinar unit aids the expulsion of saliva from acinar cells into the ductal network, composed of intercalated, striated, and excretory ducts, through which saliva is modified and transported to the oral cavity (Figure created with BioRender.com).

Salivary glands are composed of two types of secretory acinar cells surrounded by myoepithelial cells that help the secretion of the mucous or serous fluid into the ductal network through which saliva reaches the oral cavity (Fig. 1)^26–28^.

During salivary gland homeostasis, a single administration of 3H-thymidine labeled intercalated ducts and to a lower extent acinar cells and granulated ducts^29^. Over time, the number of labeled intercalated ductal cells decreased, while the number of labeled acinar and granulated ductal cells increased, potentially identifying the intercalated ductal cells as candidates for transient amplifying (T/A) progenitor cells^29–31^. Although the intercalated duct seems to be recognized as the T/A compartment in salivary glands agreeing with the unidirectionality of the differentiation stream proposed based on the model of HSCs, the identity of the multipotent cell that occupies the apex of the salivary gland stem cell hierarchy tree remains unknown.

While LRC studies in adult salivary gland show a scattered distribution of LRCs throughout the parenchyma and co-expression of putative salivary gland progenitor cell markers^32^, these studies only focus on the gland in homeostatic situations and do not consider the limitations that arise using a label retaining approach. LRC studies are unable to discriminate between potential quiescent stem/progenitor cells and other cell types which are cycling slowly at the moment of the pulse or differentiated cells that have ceased dividing and thus potentially generate false positives. In order to address the nature and regenerative potential of LRCs, a label retaining approach should be combined with an injury model to verify whether the number of LRCs stays the same or decreases and the percentage of proliferating LRCs and whether these proliferating LRCs (if present) contribute to salivary gland regeneration in a multipotent way, giving rise to both acinar and ductal cells. Currently, label retaining approaches applied to adult salivary glands have resulted in being neither sensitive enough nor specific enough^32^ for the identification of salivary gland stem/progenitor cells, and it is therefore not possible to conclude, based on their spatial localization and cycling characteristics, whether salivary gland LRCs are (or are not) stem cells.

**Fig. 2:**
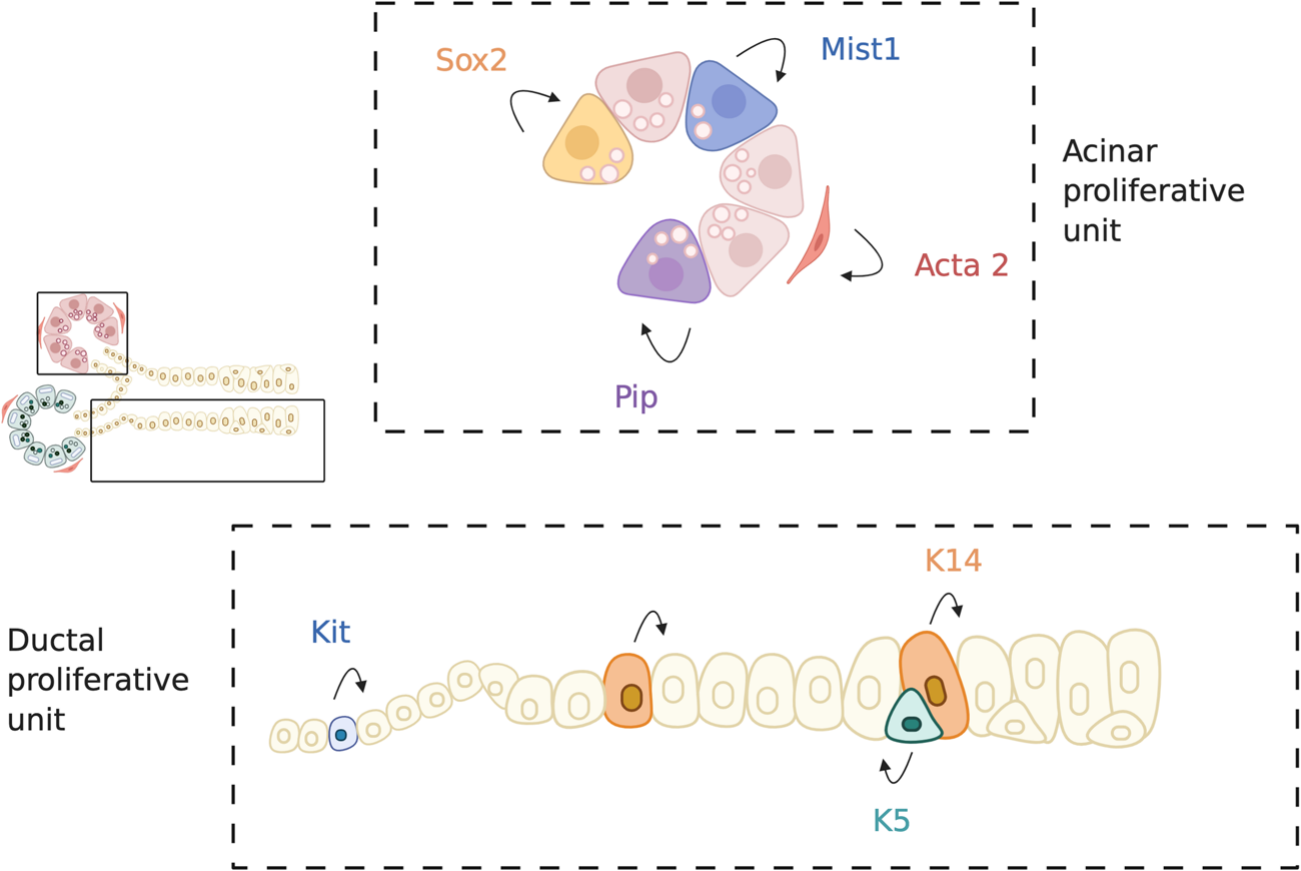
Salivary gland homeostasis seems to be achieved via self-duplication of lineage-restricted progenitor cells. Salivary glands are suggested to be structured in salivary gland proliferative units (SPUs), respectively, the acinar and the ductal proliferative units. Each unit contains multiple lineage-restricted progenitor cells, which, under normal homeostatic conditions, are able to self-duplicate and replace the lost cells within the unit (Figure created with BioRender.com).

The recent use of genetic lineage-tracing models in salivary gland has provided new insights into the nature and properties of adult tissue progenitor cells. Tracing of adult acinar cell markers or markers for acinar progenitors, such as Mist1, Pip, and Sox2, revealed that homeostasis of the acinar compartment can be achieved via self-duplication of acinar cells or the replacement of mature acinar cells by immature acinar progenitor cells^33–35^ without the contribution of a more primitive adult stem cell population. Keratin-14 (K14), Keratin-5 (K5), and Kit all mark different cell types within the ductal compartment and act as lineage-restricted progenitors to maintain the ductal compartment during homeostasis^36–38^. Moreover, lineage tracing for the myoepithelial marker Acta2 (alpha-smooth muscle actin) proves that myoepithelial cells are maintained through self-duplication^36^. In contrast to adult homeostasis, stem/progenitor cells identified during embryonic development are more multipotent and less lineage restricted. For example, K5 and Sox2 are co-expressed throughout cells of the oral epithelium prior to salivary gland development^39^ and mark a population of cells that give rise to all epithelial cells of the submandibular and the sublingual glands^40,41^. However, this multipotent cell population becomes restricted to cells of the ductal and acinar lineages, respectively, as development progresses^34,36^. This evidence points to the transition from a multipotent state during development to distinct, unipotent salivary gland proliferative units in adulthood that provide lineage-restricted support to their compartment of origin in homeostatic conditions (Fig. 2). In contrast to HSCs, these cells are relatively abundant, they are not quiescent, they mostly seem to divide symmetrically, and their persistence in the tissue is subjected to stochastic events. Therefore, when comparing salivary gland stem/progenitor cells to the paradigm of the HSCs, we have to face the reality that, so far, we do not know where or, more importantly, if a multipotent “professional” quiescent stem cell exists within the salivary glands. Could it then be that salivary glands do not contain such stem cell types but rely entirely on the proliferative capacity of the three main differentiated cell types: acinar, ductal, and myoepithelial cells, and could this be explained by the tissue’s development?

**Fig. 3:**
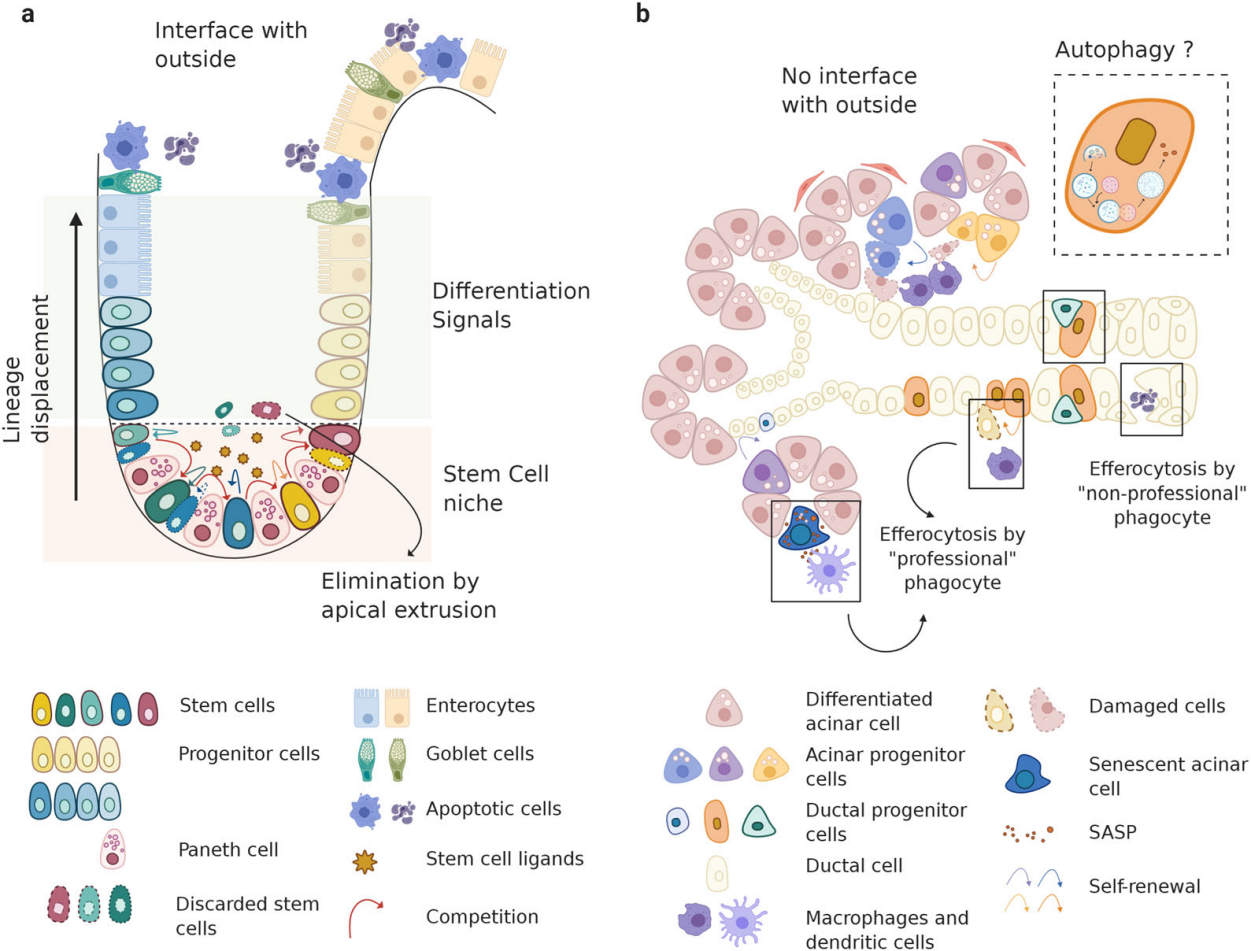
Tissue anatomy and niche size determine the regenerative strategy adopted by different tissues. Adult stem cells in fast turnover epithelial tissues, such as the intestine (**a**), are subjected to neutral competition for limited stem cell-promoting factors and niche space. Only cells that have the capacity to receive stem cell-promoting factors will remain stem cells, the others will be eliminated by extrusion from the niche or via lineage displacement in the outer space. In a slow turnover tissue, such as the salivary gland (**b**), the high cellular density, as well as the branching structure, of the tissue could explain the absence of a “professional” stem-like cell and explain instead the presence of lineage-restricted progenitors. The absence of a direct opening for disposal of damaged/unwanted cells could force salivary glands to use alternative slower clearance routes, such as autophagy or tissue-resident immune cells (Figure created with BioRender.com).

Tissues with a fast turnover, such as epidermis or intestine, contain cells with a lifetime of days or weeks, and while they are more easily exposed to insult because of their proximity to the external environment, their natural morphology allows a fast disposal route of cell debris: a direct interface with the outside world^42^. Lineage-tracing studies and statistical analysis in fast turnover tissues, such as the testis^43^, intestine^44,45^, esophagus^46^, and glandular stomach^47^, revealed that each of these tissues are devoid of slow cycling stem cells, which are central to the dogma of the HSCs. In contrast, they appear to possess a pool of equipotent proliferating stem-like cells (clones), which are capable of giving rise to all differentiated cell lineages in the tissue. All these equally potent stem-like cells compete with each other for niche space and their long-term permanence in the niche will depend solely on stochastic events^48,49^, similar to what is described as the neutral theory of molecular evolution in population genetics^50^. In adult tissues, the heterogeneity of the niche structures, the niche size, and the variety and spatial distribution of the signals released from the niche in relation to stem-like cell location could drive stochastic cell fate decisions. Hence, following a neutral drift dynamic, only certain clones persist in the niche. All other clones are displaced and pushed away from stem cell-promoting factors by their actively dividing neighbors, are exposed to differentiation factors, and ultimately are cleared into the external environment^45,49,51,52^ (Fig. 3a). This continuous process of rapid division and disposal of unwanted/used or damaged cells mostly by cell extrusion shapes these tissues, constantly maintaining the appropriate balance for a correct tissue homeostasis^53^.

On the other hand, when tissues with a slow turnover, such as the salivary gland, liver, lung, or prostate, are considered, one could hypothesize that the cellular density of these tissues and the heterogeneity of the epithelial composition throughout the tissue, as well as the absence of a direct external environmental interface, could evolutionarily explain the potential “absence” of a single and spatially segregated, quiescent multipotent stem cell population, as well as the presence of multiple populations of proliferative stem/progenitor-like cells, in these tissues. While a quiescent multipotent stem cell population spatially segregated in the tissue (for example, in the basal layer of the main excretory ducts) may result in an inefficient short-term regeneration strategy due to the physical distance imposed by the branched morphogenesis of the tissue, other strategies such as plasticity of differentiated cells could account for rapid tissue repair^54^. The absence of a direct disposal route could be the reason why these slow turnover organs invested in a long-term maintenance approach, where the balance between new cells and the clearance of senescent cells and cellular debris could potentially require more sophisticated routes, like resident macrophages^55^. This could involve, for example, efferocytosis, potentially performed by tissue-resident “professional” (macrophages, dendritic cells) or “non-professional” (epithelial cells) phagocytes, or autophagy^56^ (Fig. 3b).

## Regenerative cells: “the usual suspects” or “shape-shifters”?

Despite its slow turnover and the current lack of proof for the existence of a multipotent stem cell, studies on salivary gland damage have revealed a great regenerative potential of the gland, which varies depending on the type of damage inflicted. Upon ligation of the main salivary gland excretory duct, the acinar parenchyma is drastically reduced^33^. Lineage-tracing studies revealed that, following removal of the ligation, the few remaining acinar cells in the damaged gland re-enter the cell cycle and begin cell division without de-differentiating to a stem-like state and subsequently drive regeneration of the gland. Within 7–14 days, the acinar tissue of the gland is completely restored^33^. While mild and reversible damage to the gland, such as the described ligation-induced damage, appear to be rescued in a lineage-restricted manner, an alternative repair mechanism seems to come into play when broader stressors, such as radiation, irreversibly damage the whole salivary gland parenchyma.

**Fig. 4:**
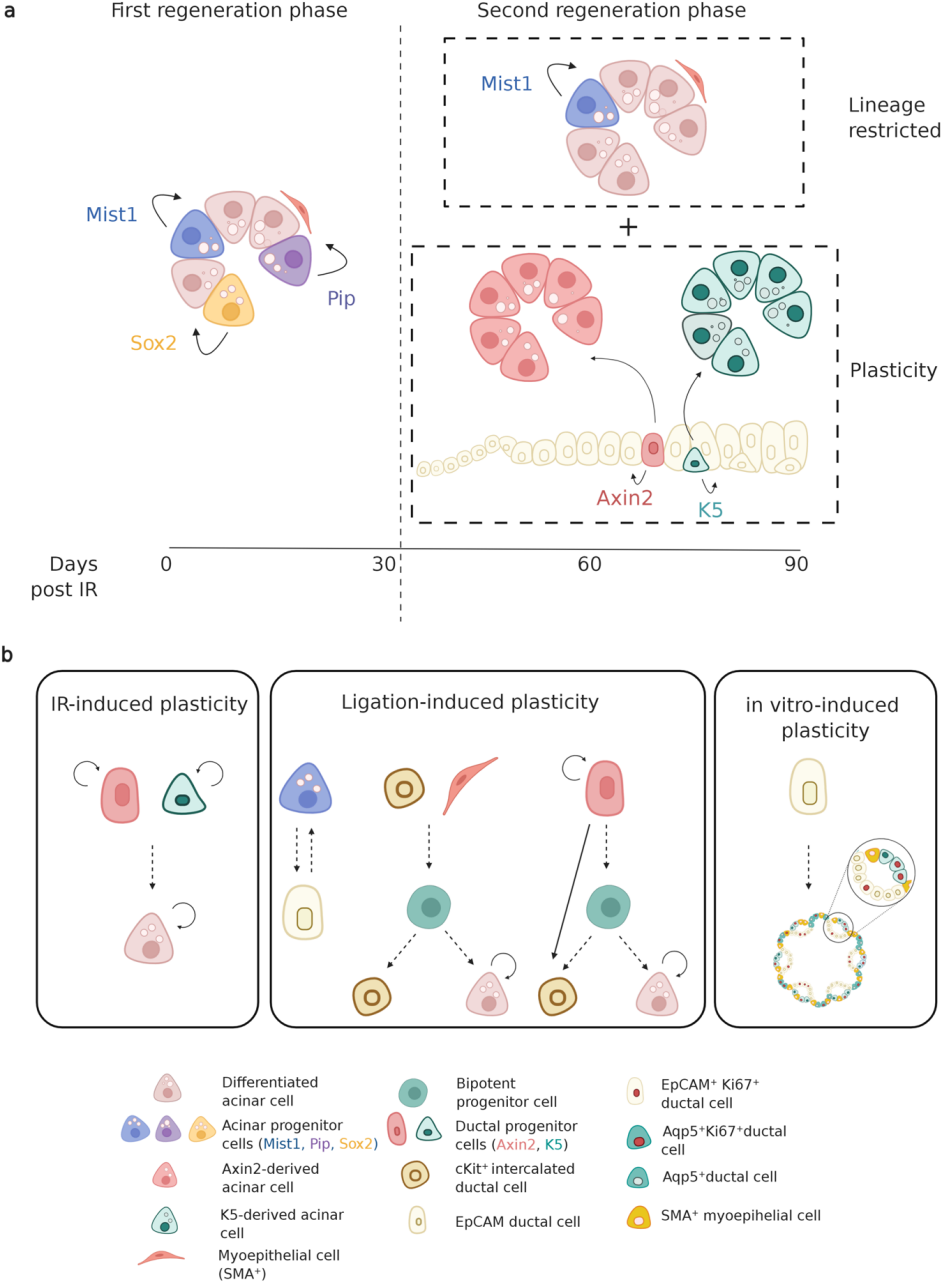
Mechanisms of plasticity after damage in salivary glands. **a** Salivary gland regeneration phases after radiation-induced damage. Upon radiation damage, a first regeneration phase seems to be characterized by a lineage-restricted response defined by the self-duplication ability of acinar cells (Mist1 and Pip) into acinar cells as well as the replacement of acinar cells driven by immature acinar progenitor-like cells (Sox2) that within the first 30 days post-irradiation are able to maintain the integrity of the acinar cell compartment. The second regeneration phase, marked by an extensive loss of acinar cells, seems to rely on the plasticity properties of the ductal cells (Axin2 and K5) that are able to give rise to new acinar cells. **b** In the context of tissue damage, salivary gland progenitor cells exhibit plasticity. Depending on the type of injury inflicted, salivary gland cells show different damage-induced plasticity: irradiation-induced plasticity, ligation-induced plasticity, and in vitro induced plasticity (giving rise to an organized 3D system formed by different cell types named organoid) (Figure created with BioRender.com).

Despite the beneficial effect of targeted tumor treatment, radiotherapy for head and neck cancers often inflicts damage to the surrounding healthy salivary gland tissue (which is often unavoidably included in the irradiation field) causing severe complications for surviving patients^57^. Analysis of clinical data to investigate the dose–volume response in salivary gland function revealed that up to 40 Gy fractionated radiation (tumor dose 60–75 Gy in 1.8–2.0 Gy fractions 5 days a week, over 5–7 weeks) salivary glands may maintain a partial regenerative capability^58–61^. While it is important to take the dose, the volume, and the effect of a single dose compared to a fractionated radiation schedule into consideration when looking at a regenerative response, murine models have been widely used to study and describe the kinetics of the salivary gland radiation-damage response^62^. A single dose of 15 Gy of X-rays, delivered locally to the glands, induces a slow and progressive decline of the acinar cell unit that culminates 90 days post local rat salivary gland irradiation^60,63^ and seems to resemble best the clinical dose response^64^. Recent lineage-tracing studies in mice indicated that, at 30 days following doses of 10–15 Gy γ-rays irradiation, when little acinar cell loss has occurred, acinar and ductal cells activate “a first regeneration response”, similar to the one described for ligation-induced damage, which can be described as a lineage-restricted regeneration response: acinar cells give rise to acinar cells^34^, while ductal cells remain restricted to replacing ductal cells^36,65^. This phase can be described as a “mild-damage phase”, considering that the parenchyma of the gland is still intact^63^. The question remains as to what extent this specific “first regenerative response” occurs after higher doses when fewer remaining cells are capable of division. The subsequent 60 days can be described instead as a “severe-damage phase”: only a few clusters of isolated acinar cells are present in the gland, while no major changes are evident in the ductal compartment^66^. Upon such conditions, Weng et al.^65^ provided proof for an in vivo response involving ductal cell plasticity. By tracing the lineage of two distinct ductal cell populations (expressing K5 and Axin2; shown in Fig. 4a, b), they demonstrated that 90 days post 15 Gy γ-ray irradiation ductal cells rather than “a professional” stem cell population were responsible for a “second regeneration response” attempting to replace the severely compromised acinar cell compartment. Recent fate-mapping analysis upon unilateral ligation of the main excretory duct showed a remarkable plasticity of the ductal (K14^+^ and cKit^+^) and myoepithelial cells (SMA^+^) to replenish the lost secretory acinar compartment. While K14^+^ cells activate a multipotency program able to give rise to both acinar and ductal cell lineages, the main contribution to acinar cell replacement seems to take place via dedifferentiation of both myoepithelial cells and cKit^+^ cells into a common bipotent progenitor cell that gives rise to cKit^+^ cells and differentiate into acinar secretory cells^67^ (Fig. 4B). Recent studies revealed that it is not only ductal cells that can acquire a multipotent stem-like state but also acinar cells have been suggested to respond to ligation-induced injury by undergoing acinar-to-ductal metaplasia^68^, similar to the response known to be required for the survival of pancreatic acinar cells subjected to stress^69^. It would be of interest to address whether, like pancreatic acinar cells, also salivary gland acinar cells possess a “protective plasticity”, the ability to re-acquire the secretory phenotype once the damage is resolved^68,69^ (Fig. 4b). While there have been only a few studies that reported the plasticity of the salivary gland epithelium during in vivo regeneration, which seems to be dependent on the type and severity of injury, radiation, or ligation, this phenomenon can be recapitulated in vitro. Clonal organoids derived from single cell-sorted salivary gland ductal epithelial cells (EpCAM^+^, epithelial cell adhesion molecule marker, or CD24^+^/CD29^+^) exhibit the capacity to proliferate and give rise to organoids containing the three major cell types present in the salivary gland when cultured in Matrigel®^8,70^. The ability of these, thought to be post-mitotic fate-restricted, cells to re-enter the cell cycle is an indication that with the appropriate signaling factors they can acquire the potential to revert to a multipotent stem-like cell state. While lineage-tracing studies would be needed to confirm in vitro (and in vivo) plasticity mechanisms of EpCAM^+^ cells, we could speculate that plasticity of these cells could occur via the activation of a “revival” cell^71^, a rare, non-regenerative cell during homeostasis that upon damage can activate a transient expansion program to reconstitute the progenitor/multipotent cell pool responsible for the generation of the salivary gland lineages, similar to the transdifferentiation process recently described for SMA^+^ and cKit^+^ cells^67^.

## Salivary gland organoids and niche signaling

The development of salivary organoid cultures has increased the knowledge of how to control and characterize the behavior of salivary gland-derived cells, such as the ability to proliferate and to differentiate into distinct lineages in terms of marker expression and at a molecular level^8,70^. The optimization of culture conditions^8^ and the three-dimensional support of an extracellular matrix^70^, as well as an increased effort to reproduce in vitro the biochemical signals produced by the native microenvironment^72^, contributed to their huge expansion potential that has opened up the possibility for using salivary gland-derived organoids as a source for cell therapies. Transplantation of salivary gland organoid-derived cells into irradiated murine salivary gland resulted in engraftment, viability, and long-term survival of the transplanted cells into the host. Selection, expansion, and transplantation of salivary gland stem/progenitor cells based on molecular markers taken from well-characterized stem cell systems, such as that of the mammary gland (CD24/CD29)^73^, indicated their ability to rescue the irradiation-induced hyposalivation up to 50% compared to non-transplanted irradiated animals^70^. In salivary gland, the CD24 and CD29 markers label all the ductal cells in the gland, although with different expression intensities between cells^70^, raising the possibility that a population of cells with stem-like characteristics could be hidden under the umbrella of a mature lineage.

While the niche signaling that regulates homeostasis in the adult gland remains poorly understood, lineage-tracing studies of Axin2-Cre Wnt-responsive cells have shown restricted Wnt activity in the ductal compartment^65^. Moreover, basal EpCAM^+^ ductal cells co-express nuclear β-catenin, an intracellular signal transducer in the Wnt signaling pathway, most abundantly in the excretory ducts^8^. Organoid culturing of EpCAM^+^ ductal cells showed an unprecedented expansion and multipotent potential upon Wnt3a and R-spondin stimulation. Wnt-derived organoids facilitated the engraftment and repopulation of irradiated salivary glands upon transplantation and restored 80% of the saliva secretion^8^. Contrary to the long-standing belief that we should be searching for stem cells based on the phenotypic identity of the cells, recovery in saliva production obtained with unselected Wnt-stimulated cells suggests that we should look at the functionality of the cells in terms of their ability to replace damaged tissue instead of surface markers^74^. It could be possible that, rather than the identity of the cells transplanted, the signals they are exposed to are responsible for their in vitro and in vivo regenerative potential. Alternatively, it has been suggested that, beside the niche signaling the cells are exposed to and that are responsible for their activation, transplanted activated cells could be a potential source of paracrine factors^75–77^, such as cytokines, growth factors, or extracellular vesicles, that could contribute indirectly to the final therapeutic benefit of cell transplantation^78^. Taken together, these findings showed that, as the stem cell field evolves, dogmas and definitions are becoming outdated and most mammalian adult tissues, including the salivary glands, do not follow the traditional HSC paradigm. Instead, it seems that they display more plasticity that was previously believed, with the response of the tissue depending on the type and intensity of the damage.

## Future directions and therapeutic perspectives

While in fast turnover tissues, such as the intestine, stem cell function can be distributed over a large population of cycling cells that compete for niche space, slow turnover epithelial tissues appear to adopt a different repair strategy. In the salivary glands as well as in the pancreas^79–81^ and liver^82^, stem cell function may be executed through the plasticity of previously thought to be terminally differentiated cells. Plasticity in the salivary gland could involve a switch in cellular identity from one differentiated cell to another type of differentiated cell as described upon ligation injury. Alternatively, it could be executed by “revival” cells, being rare terminally differentiated cells activated upon injury that are able to replace the progenitor cell pool responsible for the regeneration of the damaged tissue. This is suggested to occur in the second regeneration phase of irradiated salivary gland, upon unilateral ligation of the main duct and potentially during organoid culture, similarly to what has been observed in the liver and intestine^71,82^. A change in stem cell definition and the phenomenon of plasticity bring questions regarding the meaning of what is always referred as terminal differentiation. What was once described as a unidirectional process along a hierarchical tree toward a final state appears now as a dynamic process where several steps along the route may be reversible. Every nucleated cell in our body possesses the same genetic information and therefore the potential to change phenotype. Could we then argue that, more than nature, here meant to be a fixed set of genes or specific surface markers, it is nurture that will define the state of cells with regards to their ability to execute stem cell function and replenish lost or damaged tissue? How should we then characterize the regeneration process of an adult tissue, such as the salivary glands, where the stem cell hierarchy is unknown and where plasticity more than phenotypic characteristics could be responsible?

In vivo lineage-tracing studies have so far shed some light on salivary gland cell fate decisions during homeostasis and regeneration in model organisms, such as mice. However, these types of genetic manipulations are not feasible in the study of human tissue regeneration processes. While the expression of murine cell markers could be used as a starting point to explore the regeneration ability of the human salivary gland, it has been shown that those markers are either not expressed or are not equally potent in human tissue^1^. Culturing of unselected human salivary gland-derived cells as organoids and the transplantation of these cells into locally irradiated salivary glands exploits the regenerative potential of these cells^75^.

New, broader, and unbiased approaches, which do not require prior knowledge of genes and markers expressed by a cell of interest, are therefore needed to unravel cellular lineage relationships during adult salivary gland tissue regeneration as well as to investigate the niche signals responsible for lineage conversion of both murine and human salivary gland cells. While the niche of two species may differ, xeno-transplantation remains the closest available model to study the functionality and fate of salivary gland-derived cells. However, achieving single-cell resolution to identify the regenerative potential of a specific cell type might be complicated. A combination of organoid culture and heritable DNA barcodes, introduced at a single-cell level and read using next generation sequencing, could allow one to perform clonal analysis and lineage tree reconstruction, identifying the potency of salivary gland derived cells, without the need for a starting identity. The advancement of single-cell RNA sequencing, the principle of which is based on the assumption that cells that are genetically closely related have similar transcriptomes, and their algorithm of analysis could be of use in salivary gland regeneration studies to capture cellular lineage relationships as well as to probe cellular composition and dynamics of the niche under different conditions.

Recent evidence has shown that environmental changes can induce epigenetic modifications of chromatin that can alter stem/progenitor cell behavior providing the plasticity necessary to adapt to the changing environment^83^. Currently, there is very little knowledge on the salivary gland epigenetic landscape. How it is set, maintained, and regulated in terms of DNA methylation, histone modification, and chromatin remodeling via pioneer transcription factors during salivary gland development and regeneration remains unknown. These epigenetic events could be responsible for controlling the transcriptomic switches that determine cell-fate decisions, as well as plasticity, of specific cell types during development and regeneration. A deeper understanding of these events in the salivary glands could lead to the identification of specific molecular cell state(s) that could potentially be used for drug targeting to stimulate endogenous regeneration of damaged glands and promote the development of cell plasticity-based regenerative therapy^84^. Furthermore, identifying the underlying epigenetic mechanisms triggered upon injury to induce cell plasticity could allow the further development of epigenetic engineering approaches in cellular model systems, such as salivary gland-derived organoids. This could enhance or potentiate the already proven potential of regenerative therapies to rescue the radiation-induced hyposalivation phenotype.

Each cell in every organ/tissue of the adult body has evolved different cues to meet the tissue-specific requirement, including that of regeneration and repair. From this review, it is apparent that salivary gland stem cells are not embodied in a HSC-like hardwired “professional” stem cell system but likely are “facultative” stem-like cells^85^ that play a role in the regenerative response of the salivary glands. While at present in vivo gene therapy approaches provide encouraging results^86,87^ in the treatment of salivary gland dysfunction, the characterization of a stem cell-like functional state would open the possibility to combine ex vivo stem cells and gene delivery to create an “optimal cell population.” This approach would guarantee both the incorporation of the functional replacement gene(s) into the host DNA and the ability to ensure that the new information would be permanently part of the new cell population of the tissue, thus avoiding current problems with vector incompatibility, transduction efficiency, and short-term gene expression. Looking into the future, taking advantage of computational modeling to integrate multiomics single-cell data to understand cell–cell interaction dynamics and the behavior through time of cell populations within the regenerating salivary gland could be a unique opportunity to unravel cell fate transition in salivary gland tissue regeneration and the pathways by which they are specified. This could lead to the possibility of pharmacologically controlling and/or stabilizing a specific cellular state. Ultimately, these approaches could subsequently be used as therapeutic strategy to rescue radiation-induced hyposalivation and improve the quality of life of many thousand head and neck cancer patients.
